# Crystal Structure of Interleukin-6 in Complex with a Modified Nucleic Acid Ligand

**DOI:** 10.1074/jbc.M113.532697

**Published:** 2014-01-12

**Authors:** Amy D. Gelinas, Douglas R. Davies, Thomas E. Edwards, John C. Rohloff, Jeffrey D. Carter, Chi Zhang, Shashi Gupta, Yuichi Ishikawa, Masao Hirota, Yuichiro Nakaishi, Thale C. Jarvis, Nebojsa Janjic

**Affiliations:** From the ‡ SomaLogic, Inc., Boulder, Colorado 80301,; § Emerald Bio, Bainbridge Island, Washington 98110, and; the ¶ Otsuka Pharmaceutical Co., Ltd., 463-10 Kagasuno, Kawauchi-cho, Tokushima 771-0192, Japan

**Keywords:** Aptamers, DNA Structure, Drug Discovery, Interleukin, Molecular Evolution, Nucleic Acid Chemistry, Protein-Nucleic Acid Interaction, G-quartet, SELEX

## Abstract

IL-6 is a secreted cytokine that functions through binding two cell surface receptors, IL-6Rα and gp130. Because of its involvement in the progression of several chronic inflammatory diseases, IL-6 is a target of pharmacologic interest. We have recently identified a novel class of ligands called SOMAmers (S low Off-rate Modified Aptamers) that bind IL-6 and inhibit its biologic activity. SOMAmers exploit the chemical diversity of protein-like side chains assembled on flexible nucleic acid scaffolds, resulting in an expanded repertoire of intra- and intermolecular interactions not achievable with conventional aptamers. Here, we report the co-crystal structure of a high affinity SOMAmer (*K_d_* = 0.20 nm) modified at the 5-position of deoxyuridine in a complex with IL-6. The SOMAmer, comprised of a G-quartet domain and a stem-loop domain, engages IL-6 in a clamp-like manner over an extended surface exhibiting close shape complementarity with the protein. The interface is characterized by substantial hydrophobic interactions overlapping the binding surfaces of the IL-6Rα and gp130 receptors. The G-quartet domain retains considerable binding activity as a disconnected autonomous fragment (*K_d_* = 270 nm). A single substitution from our diversely modified nucleotide library leads to a 37-fold enhancement in binding affinity of the G-quartet fragment (*K_d_* = 7.4 nm). The ability to probe ligand surfaces in this manner is a powerful tool in the development of new therapeutic reagents with improved pharmacologic properties. The SOMAmer·IL-6 structure also expands our understanding of the diverse structural motifs achievable with modified nucleic acid libraries and elucidates the nature with which these unique ligands interact with their protein targets.

## Teaser

**Background:** Traditional aptamers favor polar interactions with protein binding partners.

**Results:** The IL-6·SOMAmer structure reveals an interface rich in hydrophobic interactions that overlap the binding sites of IL-6 receptors.

**Conclusion:** Hydrophobic modifications on DNA scaffolds generate diverse and novel structural motifs.

**Significance:** Synthetic SOMAmers are potent, specific, and chemically versatile ligands with distinct binding properties compared with conventional aptamers.

## Introduction

Interleukin-6 (IL-6) is a major contributor to the progression of many chronic inflammatory diseases and a well-established target for pharmacologic intervention (1, 2). Mammalian IL-6 is a secreted cytokine that exerts its biologic effects through binding to two cell surface receptors, IL-6Rα and gp130 (3–5). The assembly of the IL-6·IL-6Rα·gp130 ternary complex on the cell surface activates the JAK family of tyrosine kinases and the downstream STAT3 transcription factor (6–8). In cells that express IL-6Rα, IL-6-dependent activation of the signal-transducing gp130 receptor is mediated by membrane-bound IL-6Rα (*cis*-signaling). Alternatively, IL-6 in a complex with soluble IL-6Rα (sIL-6Rα) can activate gp130 in cells (*trans-*signaling) (9). In the accompanying paper (36), we report the identification of two distinct classes of SOMAmers (Slow Off-rate Modified Aptamers) that bind IL-6 with subnanomolar affinity and in a manner that interferes potently with IL-6-mediated signaling. SOMAmers are distinguished from conventional aptamers in that they contain modified side chains that enhance functional group diversity of nucleic acids and thereby facilitate selection of ligands with exceptional thermodynamic and kinetic stability reflected in very tight binding and slow dissociation rates (10, 11). These side chains can be introduced at positions that do not interfere with base-pairing (and are therefore compatible with enzymatic steps of SELEX^3^), with the 5-position of pyrimidines representing a suitable position for such base modifications (11). In general, the use of modified DNA libraries with increased hydrophobic character has dramatically improved the success rate of SELEX (10). The accompanying paper (36) describes IL-6 SOMAmers identified from two randomized modified DNA libraries, each with one modified nucleotide: 5-(*N*-benzylcarboxamide)-2′-deoxyuridine (Bn-dU) or 5-[*N*-(1-naphthylmethyl)carboxamide]-2′-deoxyuridine (Nap-dU) (36).

Here, we report the three-dimensional structure of the Bn-dU SOMAmer in a complex with IL-6 at a resolution of 2.4 Å. The co-crystal structure reveals a large binding interface characterized by extensive shape complementarity between the binding partners in which the SOMAmer engages IL-6 through two distinct domains, a stem-loop domain and a G-quartet domain. The structure also reveals the critical role of modified nucleotides in shaping the SOMAmer surface and creating hydrophobic contacts with the protein. The SOMAmer·IL-6 binding interface overlaps with the contact zones between IL-6 and its two cell surface receptors, IL-6Rα and gp130, thereby providing a compelling rationale for the potent inhibitory activity of the SOMAmer against IL-6-mediated signaling.

We have previously reported one other co-crystal structure of a SOMAmer bound to its target, platelet-derived growth factor BB (PDGF-BB) (12). The two structures exhibit certain common elements, including extensive utilization of hydrophobic interactions facilitated by the aromatic rings of the modified nucleotides. These hydrophobic networks represent a key architectural element that sets SOMAmers apart from conventional aptamers. They enable SOMAmers to adopt binding modes that mimic the highly diverse and exquisitely specific interactions typically found in protein. The IL-6·SOMAmer co-crystal structure also reveals unique binding elements and structural motifs not found in the PDGF SOMAmer, highlighting the diverse inter- and intramolecular interactions that are possible when SELEX technology is bolstered by richly varied modified nucleotide libraries.

## EXPERIMENTAL PROCEDURES

### 

#### 

##### SOMAmer Selection and Optimization

The SOMAmer was identified through SELEX using human IL-6 protein as the target and a modified Bn-dU DNA library, as described in the accompanying paper (36). Further truncation and post-SELEX modifications of chemically synthesized SOMAmer variants from the original SELEX pool resulted in SL1025 being chosen for detailed crystallographic studies. SL1025 was synthesized for crystallography studies at 50 μmol scale and was purified by HPLC.

##### SOMAmer Synthesis

SOMAmers were synthesized according to established solid-phase synthesis protocols described in the accompanying paper (36).

##### IL-6 Protein

Recombinant human IL-6 protein used in our crystallization studies (Creative BioMart, Shirley, NY; catalog no. IL6–12H) lacked the N-terminal signal peptide (mature form). Keeping with historical nomenclature, the first amino acid of the mature form was designated residue number one in these studies. The mature form of the IL-6 protein was also used in SELEX (36).

##### Analytical Size Exclusion Chromatography

The molar ratio of the SOMAmer SL1025·IL-6 complex was estimated using size exclusion chromatography using a TSK3000 column under control of a 1100 HPLC system (Agilent Technologies, Santa Clara, CA). Various molar ratios of protein and SOMAmer were examined, and the mobility of the species containing SOMAmer was measured by comparing UV absorption peaks at 280 nm to gel filtration standards (Bio-Rad). A faster migrating species, consistent with unbound SOMAmer, appeared when the molar ratio of SOMAmer/protein exceeded 1:1.

##### IL-6 Complex Formation with SL1025

SOMAmer SL1025 was combined with IL-6 at a slight molar excess (1.1:1) and diluted with SOMAmer annealing buffer (10 mm HEPES (pH 7.5), 110 mm NaCl). The mixture was concentrated in a centrifugal filter with a 10-kDa molecular mass cutoff (EMD Millipore, Billerica, MA). The complex was concentrated to ∼6.5 mg/ml of protein, as estimated by monitoring the increase in the UV absorbance signal at 260 nm from the SOMAmer component. Concentrated IL-6·SOMAmer complex was dispensed into 50-μl aliquots in thin-walled PCR tubes and flash-frozen by plunging in liquid nitrogen.

##### Crystallization of IL-6·SL1025 Complex

Aliquots of IL-6·SOMAmer complex were frozen for storage and subsequently thawed and set up for sitting drop vapor diffusion crystallization experiments by adding 0.4 μl of reservoir solution to 0.4 μl of protein·SOMAmer complex solution in the drop pedestal of a Compact Junior crystallization plate (Emerald Bio, Bainbridge Island, WA). The initial crystallization hit for the IL-6·SL1025 complex was from a condition containing 25% (w/v) PEG 3350, 100 mm BisTris (pH 5.5), and 200 mm Li_2_SO_4_. Crystals were optimized for size and diffraction quality by systematically varying PEG, buffer, pH, and salt using commercial and custom-made optimization screens. The best crystals were obtained from crystallization solutions that consisted of 31% (w/v) PEG 3350, 100 mm sodium acetate (NaOAc (pH 5.5)), and a variety of sulfate and nitrate salts in the range of 200–400 mm (*e.g.* LiNO_3_, MgSO_4_, and CsSO_4_). Optimized crystals typically had a hexagonal rod-like habit and grew to be over 300 μm in length and 50–100 μm wide. Crystals were cryoprotected and frozen for data collection by increasing the concentration of PEG 3350 to greater than 45% (w/v) and directly plunging into liquid nitrogen in a cryo-loop.

##### Data Collection and Structure Solution

Two native data sets were collected that provided the basis of the two refined IL-6·SOMAmer crystal structures. The form 1 crystal was grown from 31% PEG 3350, 180 mm LiNO_3_, 100 mm NaOAc (pH 5.5), and 2.5% (w/v) hexamine cobalt chloride, belonged to space group *P*3_2_, and had unit cell dimensions of *a* = *b* = 50.23 Å and *c* = 103.63 Å. The form 2 crystal was grown from 31% PEG 3350, 90 mm MgSO_4_, 90 mm NaOAc (pH 5.5), and 100 mm LiCl, also belonged to space group *P*3_2_ but had unit cell dimensions of *a* = *b* = 69.02 Å and *c* = 108.47 Å. The distinction between form 1 and form 2 crystals is that the larger form 2 unit cell accommodates two IL-6·SOMAmer complexes per asymmetric unit, whereas form 1 only contains one heterodimer per asymmetric unit. Both native data sets could readily be solved by molecular replacement using the program Phaser in the CCP4 software suite using IL-6 PDB coordinates (chain B of PDB code 1P9M) as a search model. However, the contribution of the IL-6 model alone was not sufficient to provide enough phasing power to elucidate interpretable maps of the electron density for the bound SOMAmer.

To obtain additional phasing information, crystals were soaked with iodide and cesium salts for the purpose of conducting single wavelength anomalous dispersion (SAD) experiments. Iodide and cesium ions have nearly identical anomalous scattering properties, give strong anomalous signals (especially at the relatively low energy produced by in-house x-ray equipment), and can be readily soaked into most types of protein crystals. Full data sets on iodide-soaked and cesium-soaked crystals were collected to ∼3 Å and used to confirm that each ion could provide several partially occupied ion-binding sites and a sizable anomalous signal. For structure solution, a crystal soaked for 20 min in crystallization reservoir solution supplemented with 500 mm LiI and 500 mm CsCl was examined at ALS beamline 5.0.3 (Berkeley, CA) on December 21, 2010. The wavelength of the x-rays used to collect the data was 0.9765 Å. The data were scaled to a resolution of 2.4 Å. Anomalous difference maps were calculated from the data to locate the positions of bound heavy atoms. Twelve potential sites were identified by this method. The heavy atom positions were then input, along with the IL-6 molecular replacement search model, into the program Phaser, using the “SAD plus MR” mode. This program used phasing information calculated from heavy atom positions and phasing information from the molecular replacement model to calculate phases and electron density maps. The SAD plus MR maps provided a clearer view of a portion of the SOMAmer and allowed the building of the model of the SOMAmer to begin.

Following the initial phasing of the structure, a process of “bootstrapping” was undertaken, whereby two or three nucleotide residues were built into the model, and then the larger, improved model was used as the input model for the SAD plus MR algorithms of Phaser. In each successive round, the improved MR model improved phases and thereby provided clearer electron density maps. This process continued until ∼15 nucleotide residues were built, at which time successive rounds of refinement and molecular replacement seemed to provide no improvement to the electron density maps (Fig. 10*D*). At this point, the partial IL-6·SOMAmer complex model was moved into the 2.4 Å native data set and used for simple molecular replacement. Because the 2.4 Å “form 1” native data set was higher quality data (with an *R*_merge_ more than 2% lower than for the CsI soak), the resulting electron density maps had less noise and were easier to interpret. Model building continued with refinement against the native form 1 data until a final model was obtained. The form 1 model was then used for molecular replacement in the 2.55 Å native form 2 data set. Two residues of the SOMAmer, which were disordered in the form 1 structure, could be resolved in form 2 due to a distinct crystal packing interaction at the hairpin loop of the SOMAmer. Data collection and refinement statistics for both form 1 and form 2 structures are listed in Table 1. Structure analysis was performed using PyMol (13) and web3DNA (14).

## RESULTS

### 

#### 

##### About IL-6 Structure

The structure of IL-6 has been elucidated in both apo- and receptor-bound forms (15, 16). Full-length IL-6 is comprised of 212 amino acids with an N-terminal signal peptide of 29 amino acids and a four-helix bundle arranged in an up-up-down-down topology (16, 17). The helices are historically designated A through D, from N terminus to C terminus, and contain 20–25 residues per helix with long loops connecting the helices (18). The N-terminal 20 residues do not adopt any apparent secondary structure, and only the last 7 residues of this flexible tail are discerned in the crystal structure. There is also a fifth short helix of 12 residues (amino acids 141–152) present in the long loop between helices C and D, a common feature in the long chain family of four-helix bundle proteins (19). Following this short helix, the remaining residues of the C-D loop (amino acids 131–140) are disordered and not visible in the crystal structure. Likewise, the long A-B loop (amino acids 43–79) contains 17 unresolved residues (amino acids 44–60).

##### Crystallization Studies with the SOMAmer

**FIGURE 1.**
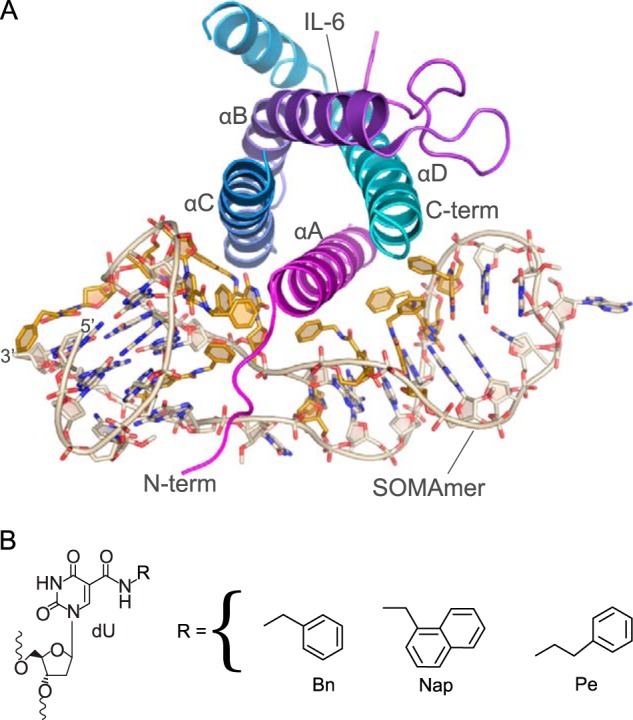
**2.55 Å crystal structure of SOMAmer SL1025 bound to human IL-6 (form 2 chains A and B).** *A,* helices of IL-6 are labeled (*A–D*) from N to C terminus (*term*) and are colored *magenta* (helix A), *purple* (helix B), *blue* (helix C), and *cyan* (helix D). The modified nucleotides of the SOMAmer are colored *gold*. This color scheme is maintained throughout the figures. *B,* chemical structures of the C-5 modified deoxyuridines present in SOMAmer SL1025.

**FIGURE 2.**
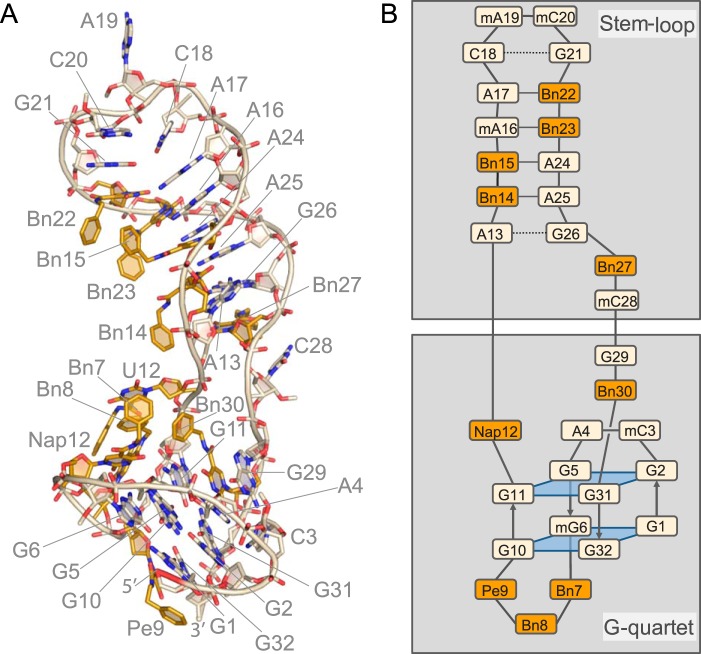
**Structure of the SOMAmer can be divided into two domains.** Domain 1 contains a G-quartet motif, and domain 2 has a stem-loop configuration. *A,* stick/cartoon view of SOMAmer SL1025. Modified nucleotides are colored *gold*. The modified nucleotide side chains are designated Bn7, Bn8, Pe9, Nap12, Bn14, Bn15, Bn22, Bn23, Bn27 and Bn30. The deoxyuridines of modified nucleotides are designated U, *e.g.* U12. This nomenclature is used throughout the figures except where noted. *B,* schematic of the SL1025 SOMAmer showing backbone trace, base pairing patterns, and the G-quartet motif seen in the co-crystal structure. Watson-Crick base pairs are depicted with a *solid line,* whereas sheared base pairs are depicted with a *dotted line*. Color scheme and approximate orientation as in *A*. The modified nucleotides are designated only by their side chain due to space constraints, *e.g. Bn7* is Bn-dU7.

For the crystallization studies reported here, we used a SOMAmer originally identified from the Bn-dU-modified DNA library (36). Crystallization trials with a limited number of other SOMAmer variants, including the Nap-dU-modified SOMAmers, did not yield crystals of sufficient size or quality for data collection. The original full-length clone, composed of 78 nucleotides, could be truncated to a 32-nucleotide sequence required for high affinity binding. Further post-SELEX modifications implemented to optimize binding affinity, inhibitory potency, and nuclease resistance led to variant SL1025 (Figs. 1 and 2) that contains six 2′-*O*-methyl substitutions, eight Bn-dU-modified nucleotides, one 5-[*N*-(phenyl-2-ethyl)carboxamide]-2′-deoxyuridine (Pe-dU), and one Nap-dU-modified nucleotide (36).

IL-6 was combined with SL1025 in solution and crystallized by sitting drop vapor diffusion (see “Experimental Procedures”). Complex formation at a 1:1 molar ratio was verified by analytical size exclusion chromatography. The crystal structure was solved by a combination of molecular replacement and heavy atom phasing methods. A partial solution was obtained by molecular replacement using the published structure of IL-6 (chain B of PDB code 1P9M (15)) as a search model. Additional independent phasing information was obtained by anomalous scattering using iodide and cesium.

We obtained two crystal structures (referred to as form 1 and form 2) of the human IL-6 protein bound to SOMAmer SL1025. Form 1 was solved to 2.40 Å and contained one IL-6 molecule and one SOMAmer molecule per asymmetric unit. Form 2 was solved to 2.55 Å and contained two molecules of IL-6 (chains A and C) and two molecules of SOMAmer (chains B and D) per asymmetric unit. The two structures are very similar overall and could be assigned to space group *P*3_2_ (see Table 1 for data collection and refinement statistics) (20). The form 1 complex can be superposed with both complexes from form 2 with a root-mean-square deviation (r.m.s.d.) of 0.431 Å spanning chains A (IL-6) and B (SOMAmer) over 898 atoms or chains C (IL-6) and D (SOMAmer) with an r.m.s.d. of 0.456 Å over 945 atoms. Similarly, the two SOMAmer molecules in form 2 align well, with an r.m.s.d. of 0.537 Å over 655 atoms. Both form 1 and form 2 structures lack the first 13–15 residues of the unstructured N terminus of IL-6 as well as residues in loop regions connecting the helices (form 1 amino acids 48–60; form 2 chain A amino acids 44–60, and chain C amino acids 44–59) and at the C terminus (amino acids 131–135 in form 1 and amino acids 131–140 in form 2 chain A and amino acids 131–135 in chain C). In addition to the disordered residues, form 1 has 24 amino acid residues where the polypeptide backbone was visible in the electron density, but there was insufficient density to support the modeling of side chains. This is comparable with the modeled structure of IL-6 in the IL-6 receptor complex structure (PDB code 1P9M), which has 28 disordered side chains in the model. In form 2, the number of disordered residues in chain A is 18 and 21 in chain C. Additionally, nucleotides 19 and 20 of the SOMAmer have no discernible electron density in form 1 but can be resolved in form 2. Because the form 1 and form 2 structures are nearly identical, the analysis reported here was done using the more complete IL-6·SOMAmer structure in form 2, specifically chains A and B. The IL-6·SOMAmer complex composed of chains A (IL-6) and B (SOMAmer) is shown in Fig. 1*A*. The SOMAmer interacts with the N- and C-terminal poles of the IL-6 four-helix bundle, wrapping around the protein perpendicularly to the long axis of the helices. The conformation of IL-6 in the SOMAmer-bound structure is essentially the same as that observed in the IL-6·IL-6Rα·gp130 hexameric structure, PDB code 1P9M (15). These two IL-6 structures can be superposed with an r.m.s.d. of 0.717 Å over 832 atoms.

**TABLE 1:** **Crystallographic data collection and refinement statistics** Values in parentheses indicate the values for the highest of 20 resolution shells. *R*_merge_ = Σ*_h_*Σ*_i_*|*I_i_*(*h*) − 〈*I*(*h*)〉|/Σ*_h_*Σ*_i_I_i_*(*h*). *R*_free_ = Σ*_h_*‖*F*_obs_| − |*F*_calc_‖/Σ*_h_*|*F*_obs_|. The free *R-*factor was calculated using 5% of the reflections omitted from the refinement (21). r.m.s.d. is root mean square deviation.

	Form 1	Form 2
**Data collection**		
Space group	*P*3_2_	*P*3_2_
Unit cell parameters: *a*, *b*, *c*	50.23, 50.23, 103.63 Å	69.02, 69.02, 108.47 Å
Wavelength	1.03318 Å	1.03318 Å
Resolution range	50.00 to 2.40 Å (2.46 to 2.40 Å)	50.00 to 2.55 Å (2.62 to 2.55 Å)
Unique reflections	11,411	18,817
Completeness	99.7% (100%)	99.7% (100%)
*R*_merge_	0.040 (0.614)	0.053 (0.522)
Mean *I*/σ (*I*)	26.9 (3.1)	22.6 (4.0)

**Refinement**		
Resolution range	50.00 to 2.40 Å (2.46 to 2.40 Å)	50.00 to 2.55 Å (2.62 to 2.55 Å)
*R*_cryst_	0.220 (0.280)	0.186 (0.252)
*R*_free_	0.260 (0.404)	0.239 (0.259)
r.m.s.d. bonds	0.010 Å	0.0080 Å
r.m.s.d. bond angles	1.92°	1.85°
Total no. of atoms	1890	3793
Wilson *B*-factor	65.61 Å^2^	67.12 Å^2^
Average *B-*factor, all atoms*^a^*	59.79 Å^2^	57.29 Å^2^
Average *B-*factor, protein atoms	59.11 Å^2^	57.11 Å^2^
Average *B*-factor, nucleic acid atoms	61.90 Å^2^	58.06 Å^2^
Disordered amino acid side chains	24	39
Residues in favored regions	139% (95.86%)	253% (90.36%)
Residues in allowed regions	6% (4.14%)	25% (8.93%)
MolProbity score (percentile)	1.91 (95th)	2.15 (93rd)
Protein Data Bank code	4NI7	4NI9

*^a^* Ligand *B*-factors are for ligands in the active sites of the protein monomers. Ligands from solvent (PEG, glycerol, etc.) were not included in the calculation.

##### SOMAmer Structure

The structures of the three types of 5-position uridine modifications and their location within the sequence of the optimized SOMAmer SL1025 are shown in Figs. 2*B* and 9*B*. All bases are in the *anti N*-glycosidic bond nucleoside conformation except G21, 2′-*O*-methyl C28, G1, G5, G10, and G31, which are in the *syn* conformation. Most of the riboses are in the C2′-endo conformation (18/32), with the remainder in C1′-exo (6/32), C3′-exo (3/32), C3′-endo (2/32), O4′-endo (2/32), and C4′-exo (1/32) conformations (Table 2). With respect to the amide bond of the modified bases, all are in the *trans* conformation with the single exception of Bn-dU22, which is in the *cis* conformation. In all of the modified bases, the carbonyl group of the C-5 linker is in the *anti* conformation with respect to the C-4 carbonyl group.

**TABLE 2:** **The list of sugar pucker conformations in the SOMAmer**

Residue	Pucker	Residue	Pucker
G1	C2′-endo	A17	C2′-endo
G2	C1′-exo	C18	C3′-endo
C3	C3′-endo	A19	C3′-exo
A4	C2′-endo	C20	C2′-endo
G5	C3′-exo	G21	C2′-endo
G6	C2′-endo	Bn-dU22	O4′-endo
Bn-dU7	C3′-exo	Bn-dU23	C4′-exo
Bn-dU8	C2′-endo	A24	C2′-endo
Pe-dU9	C2′-endo	A25	C2′-endo
G10	C2′-endo	G26	C2′-endo
G11	C2′-endo	Bn-dU27	C1′-exo
Nap-dU12	C2′-endo	C28	C2′-endo
A13	C1′-exo	G29	C2′-endo
Bn-dU14	O4′-endo	Bn-dU30	C1′-exo
Bn-dU15	C1′-exo	G31	C2′-endo
A16	C2′-endo	G32	C1′-exo

The SOMAmer can be divided into two structurally distinct domains that are essentially split along opposing surfaces of helix A, the N-terminal α-helix of IL-6 (Fig. 1). Each SOMAmer domain interacts with helix A and one other helix of IL-6; domain 1 binds to helices A and C, whereas domain 2 binds to helices A and D (Fig. 1). Domain 1 includes nucleotides 1–12 and 29–32 and forms a G-quartet motif composed of two G-tetrads as well as both the 5′ and 3′ termini. Domain 2 adopts a stem-loop structure composed of nucleotides 13–28 (Figs. 1*A* and 2).

##### Domain 1, G-quartet

**FIGURE 3.**
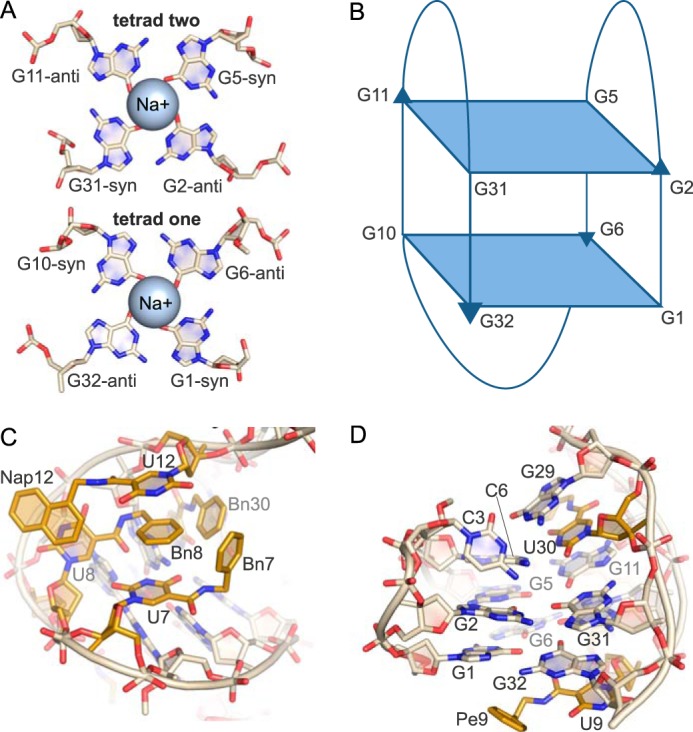
**G-quartet motif (domain 1).** *A,* G-tetrads each contain two G-bases in the *syn* and *anti* conformations. The bases hydrogen bond to neighboring G-bases through the Watson-Crick face as well as the Hoogsteen face. Each tetrad coordinates one Na^+^ ion. *B,* G-quartet conformation in the SOMAmer structure is up-up-down-down with three lateral loops. *C,* hydrophobic cluster created by modified bases Bn-dU7, Bn-dU8, Nap-dU12, and Bn-dU30. P_i_ stacking interactions occur between the uridine ring of Bn-dU7 and Nap-dU12 with Bn8. There are edge-to-face interactions between Bn8 and Bn7 and Bn30, respectively. *D,* G29 stacks with the base of Bn-dU30, which stacks with G11 of the G-quartet. The uridine ring of Pe-dU9 stacks with G32, and the modified side chain is solvent-exposed.

There are no Watson-Crick base pairs in domain 1, where the bulk of the structural integrity is derived from an intramolecular G-quartet consisting of two G-tetrads. Each G-tetrad is coordinated by a presumed sodium ion that sits in the plane of the tetrad. Tetrad one contains G1, G6, G10, and G32, and tetrad two contains G2, G5, G11, and G31 (Fig. 3*A*). Each G-quartet contains two bases in the *syn* conformation and two in the *anti* conformation. This allows each guanosine base to make two hydrogen bonds with a neighboring guanosine on the Watson-Crick face as well as on the Hoogsteen face. The sodium ions are then coordinated by the carbonyl oxygen atoms on C6 (Fig. 3*A*).

G-quartets are classified by the orientation of the strands and the glycosidic conformation. The strands in the SOMAmer G-quartet run up-down-up-down creating an anti-parallel G-tetrad core with three lateral or edgewise loops (Fig. 3*B*). Of the 26 possible topologies for three loops with contiguous G-quartet strands (21), only six have been experimentally observed (22). This specific topology was previously seen in the thrombin-binding DNA aptamer (23). Interestingly, the G-quartets of the IL-6 SOMAmer and the thrombin aptamer are structurally similar and can be superposed with an r.m.s.d. of 0.579 Å over 70 atoms. However, the IL-6 SOMAmer G-quartet domain has no binding activity for thrombin and vice versa (data not shown). In both cases, the G-quartet provides a core scaffold, whereas critical target-binding interactions derive from nucleotides in the intervening loops.

There are five modified bases in the G-quartet domain, four of which form a hydrophobic surface that contacts the protein. This hydrophobic bulge is created as a discontiguous cluster of side chains from Bn-dU7, Bn-dU8, Nap-dU12, and Bn-dU30 residues. The side chains, designated in the text as Bn7, Bn8, Nap12, and Bn30 (as opposed to Bn-dU7, which refers to the uridine ring and side chain), are brought in proximity to each other by the overall scaffold of the G-quartet in a manner that creates a series of π-stacking interactions (Fig. 3*C*). Bn-dU8 uridine ring forms a π-stacking interaction with Nap12, whereas Bn8 is sandwiched between the uridine rings of Nap-dU12 and Bn-dU7, creating additional π-stacking interactions. Bn8 is also flanked by Bn7 and Bn30, with which it forms edge-to-face π-stacking interactions, and Nap12, with which it makes edge-to-edge contact. In a sense, the Bn-dU8 nucleotide appears to serve as the core of the π-stacking hydrophobic cluster, simultaneously engaging three other modified nucleotide side chains as well as two bases. G29, which borders domain 2, stacks with the base of Bn-dU30, which in turn stacks with G11 of the G-quartet (Fig. 3*D*). The remaining modified nucleotide in this domain, Pe-dU9, does not interact with the protein but rather tucks under the G-quartet, allowing the uridine ring to stack with G32 and also with W157 of a symmetry mate, making a crystal contact. The modified side chain Pe9 is extruded into the solvent (Fig. 3*D*).

##### IL-6/SOMAmer Interactions in Domain 1

**FIGURE 4.**
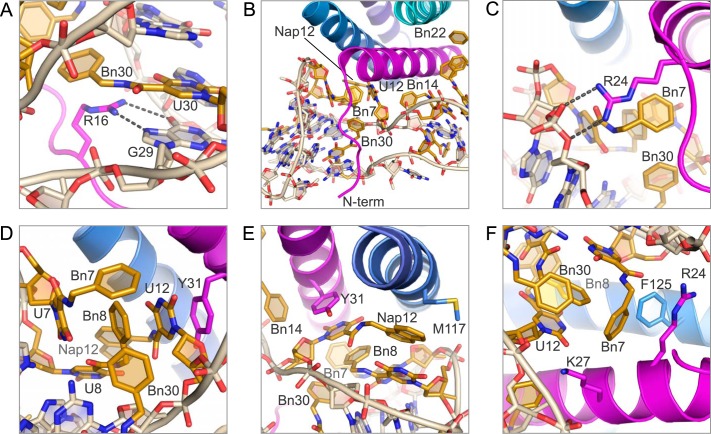
**Protein·SOMAmer interactions in domain 1.** *A,* residue Arg-16 on the IL-6 N-terminal tail hydrogen bonds to the Hoogsteen face of G29. The benzyl group of Bn-dU30 stacks against the methylene side chain of Arg-16. *B,* N-terminal tail of IL-6 is sandwiched between the backbone of the SOMAmer, protecting the hydrophobic modified nucleotides from solvent. Opposing strands of the SOMAmer backbone are in unusually close proximity to each other. *C,* Arg-24 on helix A of IL-6 forms a salt bridge to the SOMAmer backbone at G5–G6, further sealing the hydrophobic pocket from solvent. *D,* Tyr-31 on helix A of IL-6 stacks with Nap-dU12, which in turn stacks with Bn8 and the uridine ring of Bn-dU7. Bn7 and Bn30 have edge-to-face interactions with the stacked residues. *E,* Nap12 has hydrophobic interactions with the methylene side chain of Met-117 on helix C of IL-6. The naphthyl group also stacks against the uridine ring of Bn-dU8. *F,* Bn7 and Bn8 have edge-to-edge interactions with Phe-125 on helix C, and Bn7 interacts with the methylene side chains of Arg-24 and Lys-27.

Seven residues on the IL-6 protein have intermolecular contacts with domain 1 of the SOMAmer. In the N-terminal tail (residues 14–20), Arg-16 forms a hydrogen bond with G29 on the Hoogsteen face (Fig. 4*A*). Additionally, Arg-16 has hydrophobic contacts with Bn30, which stacks against the methylene groups of the arginine side chain (Fig. 4*A*). Hydrophobic intermolecular forces play a significant role in the protein/SOMAmer interactions, as seen in this repeated theme of modified nucleotides interacting with methylene side chains of amino acids (12). The N-terminal tail of the IL-6 protein lies in a pocket, created by the unusual curvature of the DNA, where it is sandwiched between three discontiguous segments of the DNA backbone. These seven unstructured amino acid residues shelter the hydrophobic cluster of Bn7, Bn8, Nap12, and Bn30 from solvent (Fig. 4*B*). The DNA backbone of the SOMAmer is distorted, creating an atypical curvature in which the phosphate groups of opposing strands are in close proximity to each other (7–10 Å) and directed toward the solvent, while the modified bases point away from the solvent, clustering together to create a protein-like hydrophobic core (Fig. 4*B*). A salt bridge between Arg-24 at the N-terminal end of IL-6 helix A and the SOMAmer backbone phosphate of nucleotide G6 imparts additional protection from solvent for the hydrophobic nucleotides (Fig. 4*C*). The modified nucleotide cluster composed of positions 7, 8, 12, and 30 clearly serves a dual function of an intramolecular structural foundation for the SOMAmer itself as well as a hydrophobic surface that contacts the protein. Tyr-31 on IL-6 helix A stacks with the uridine ring of Nap-dU12 adding the fourth aromatic ring to the string of π-stacking interactions that also involve Bn8 and the base of Bn-dU7 (Fig. 4*D*; also see Fig. 3*C*). Nap-dU12 extends to reach helix C on IL-6, where the naphthyl group participates in a hydrophobic interaction with the methylene side chain of Met-117 in addition to making a π-stacking interaction with the uridine ring of Bn-dU8 (Fig. 4*E*, also see Fig. 3*C*). Additionally, the Bn7 ring faces the methylene side chain of Arg-24 on helix A and makes an edgewise interaction with the methylene side chain of Lys-27 (Fig. 4*F*). Bn7 and Bn8 are also involved in edge-to-edge interactions with F125 on helix C of IL-6 (Fig. 4*F*).

##### Domain 2, Stem-Loop

**FIGURE 5.**
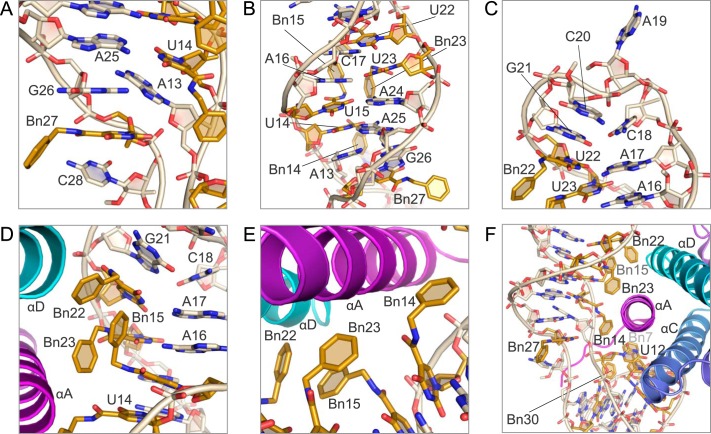
**Stem-loop motif (domain 2).** *A,* bottom of the stem loop of domain 2 contains two unpaired bases at Bn-dU27 and C28 and a sheared base pair between G26 and A13. The uridine ring of Bn27 stacks with G26; however, the benzyl group and C28 are extruded. *B,* base pairing in the stem. There are four Watson-Crick base pairs in the SOMAmer stem between Bn-dU14/A25, Bn-dU15/A24, Bn-dU23/A16, and Bn-dU22/C17. *C,* SOMAmer loop region (C18 through G21) contains two unpaired bases and a sheared base pair between C18 and G21. A19 and C20 are extruded bases. *D,* hydrophobic cluster of benzyl groups from Bn15, Bn22, and Bn23. *E,* uridine ring of Bn-dU14 stacks with the amide of Bn15, whereas the benzyl group points opposite the hydrophobic cluster of Bn15, Bn22, and Bn23. *F,* Bn-dU14 nucleotide bridges the protein interactions of domain 1 and domain 2 and interacts with hydrophobic side chains on IL-6 helix A.

Domain 2 of the SOMAmer contains a stem-loop that is primarily B-form DNA but with a slight left-handed twist in the loop region. At the bottom of the stem, on the 3′ end, are two unpaired bases, Bn-dU27 and C28. Although formally assigned to domain 2, these two unpaired bases, along with G26 and A13 on the 5′ end of the stem, can be thought of as a flexible hinge between the two domains. G26 and the uridine ring of Bn-dU27 have a weak stacking interaction, whereas the benzyl group of Bn-dU27 (Bn27) is completely solvent-exposed (Fig. 5*A*). Similarly, C28 makes no intra- or intermolecular contacts and is extruded into the solvent. These observations are consistent with the fact that Bn-dU27 can be substituted with dT, and C28 with a three-carbon (C3) spacer, without a compromise in binding affinity (36). These two unpaired bases are followed by a sheared base pair between G26 and A13 (shear, 6.2 Å; buckle, −34°; propeller, −11°) and four Watson-Crick base pairs between Bn-dU14 and A25, Bn-dU15 and A24, A16 and Bn-dU23, and A17 and Bn-dU22 that adopt B-form helix conformation (Fig. 5*B*). The Watson-Crick base pairs also exhibit a range of buckling and propeller twist parameters that deviate from ideal B-form angles with an average buckling of −14° (S.D. 26) and an average propeller twist of −11° (S.D. 9.2). The tetraloop at the top of the stem is formed with C18, A19, C20, and G21. Formally within the tetraloop, C18 and G21 form a substantially distorted base pair characterized by two stretched H-bonds (3.5 and 3.8 Å) with irregular shearing (1.1 Å), stretching (3.4 Å), stagger (1.4 Å), buckling (32°), propeller (−52°), and opening (−61°) parameters (Fig. 5*C*). The H-bonds are formed between the Watson-Crick face of C18 and the Hoogsteen edge of G21, while the Watson-Crick face of G21 is involved in a crystal contact to C20 of a symmetry mate. Additionally, the *syn* conformation of G21 results in a somewhat left-handed twist (−18°) between the A17:Bn-dU22 and the C18:G21 base pairs. The G21 base also stacks with Bn-dU22 base in the stem (Fig. 5, *C* and *D*). A19 and C20 residues are not involved in any intra- or intermolecular contacts. The C20 base is partially extruded into the solvent, while A19 is completely solvent-exposed (Fig. 5*C*).

The domain 2 stem-loop structure contains five modified benzyl nucleotides at positions 14, 15, 22, 23, and 27. Of these, only Bn-dU27 in the hinge region does not contact the protein. The remaining modified nucleotides all participate in base pairing through the uridine ring, whereas the benzyl groups are directed into the major groove of the helix and toward the protein. The protrusion of the modified groups into the major groove without disturbance to the Watson-Crick base pairs was anticipated based on similar findings with 5-methylcytosine and glucosylated 5-(hydroxymethyl) pyrimidine (24–26). However, the fact that this arrangement represents a small fraction of the observed side chain conformations could not have been anticipated, illustrating a wide repertoire of conformations accessible to 5-dU modifications for which there is no structural precedent.

A distinct hydrophobic bulge is created through the nonstacking clustered arrangement of the benzyl side chains from Bn-dU22, Bn-dU23, and Bn-dU15 (designated Bn22, Bn23, and Bn15) (Fig. 5*D*). The uridine ring of Bn-dU14 stacks with the amide linker of Bn-dU15 (Fig. 5*E*), in a similar type of intramolecular interaction involving the amide linker we observed previously (12). The benzyl group (Bn14) points away from the hydrophobic cluster comprised of Bn22, Bn23, and Bn15 but toward the IL-6 protein (Fig. 5*E*). The Bn-dU14 nucleotide bridges the protein interactions of domain 1 and domain 2 while not participating in the hydrophobic clusters from either domain (Fig. 5*F*).

##### IL-6/SOMAmer Interactions in Domain 2

**FIGURE 6.**
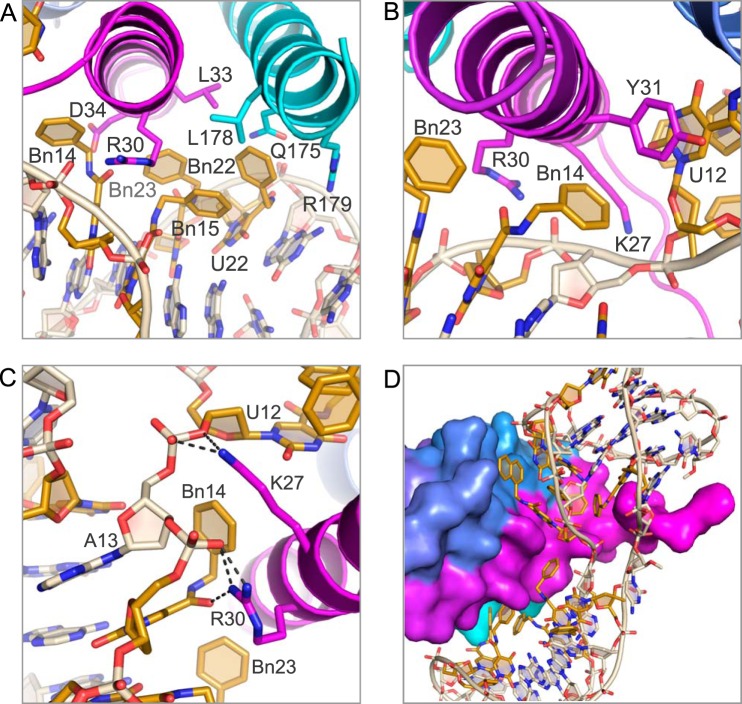
**Protein-SOMAmer contacts in domain 2 are primarily hydrophobic.** *A,* Bn15, Bn22, and Bn23 have hydrophobic interactions with the methylene side chains of residues on helix A and helix D on the IL-6 protein. *B,* Bn14 has edge-to-face interactions with Tyr-31 as well as edgewise interactions with the nonpolar side chains of Lys-27 and Arg-30. *C,* salt bridges between Lys-27 and Arg-30 on IL-6 and the SOMAmer backbone at A13 and Bn-dU14. *D,* surface rendering of IL-6 illustrates the shape complementarity of the SOMAmer·IL-6 interface.

The majority of IL-6 interactions with domain 2 of the SOMAmer are hydrophobic in nature. The benzyl groups of Bn15, Bn22, and Bn23 are nestled against helices A and D on IL-6 in a hydrophobic niche created by the nonpolar portion of the side chains of Arg-30, Leu-33, and Asp-34 on helix A and Gln-175, Leu-178, and Arg-179 on helix D (Fig. 6*A*). The benzyl group of Bn14, which is outside the aromatic cluster, has edge-to-face interactions with Tyr-31 and edgewise interactions with the methylene side chains of Lys-27 and Arg-30 (Fig. 6*B*). Two salt bridges also exist in this domain between the A13 phosphate and Lys-27, and the Bn-dU14 phosphate and Arg-30, with both amino acid residues stemming from helix A on IL-6 (Fig. 6*C*). The carbonyl oxygen of the amide linker of Bn14 also forms a hydrogen bond with the guanidinium hydrogen on Arg-30 (Fig. 6*C*). The engagement of the amide linker of the modified nucleotide in hydrogen bond formation as well as stacking interactions with other bases is a feature we have observed previously in the PDGF-B·SOMAmer co-crystal structure (12); however, this is the first observed case of a hydrogen bond between an amide linker and the protein. Thus, the amide linker of the modified nucleotides, which is conformationally constrained by the uridine ring with which it is essentially co-planar, is clearly not simply a passive spacer but rather a functional group in its own right, capable of contributing to both intra- and intermolecular interactions of SOMAmers.

A complete list of all IL-6/SOMAmer interactions is summarized in Table 3. The calculated solvent-accessible surface area (SAS) is 8696 Å^2^ for the IL-6 protein, 6672 Å^2^ for the SOMAmer, and 12,872 Å^2^ for the complex. The solvent-excluded surface area of the interface is therefore 1248 Å^2^ (calculated as ((SAS_IL-6_ + SAS_SOMAmer_) − SAS_Complex_)/2), with ∼60% of the buried surface area derived from domain 1 (Fig. 6*D*). The total interface area is similar to the previously reported solvent-excluded area of PDGF-BB SOMAmer of 1225 Å^2^ (12).

**TABLE 3:**
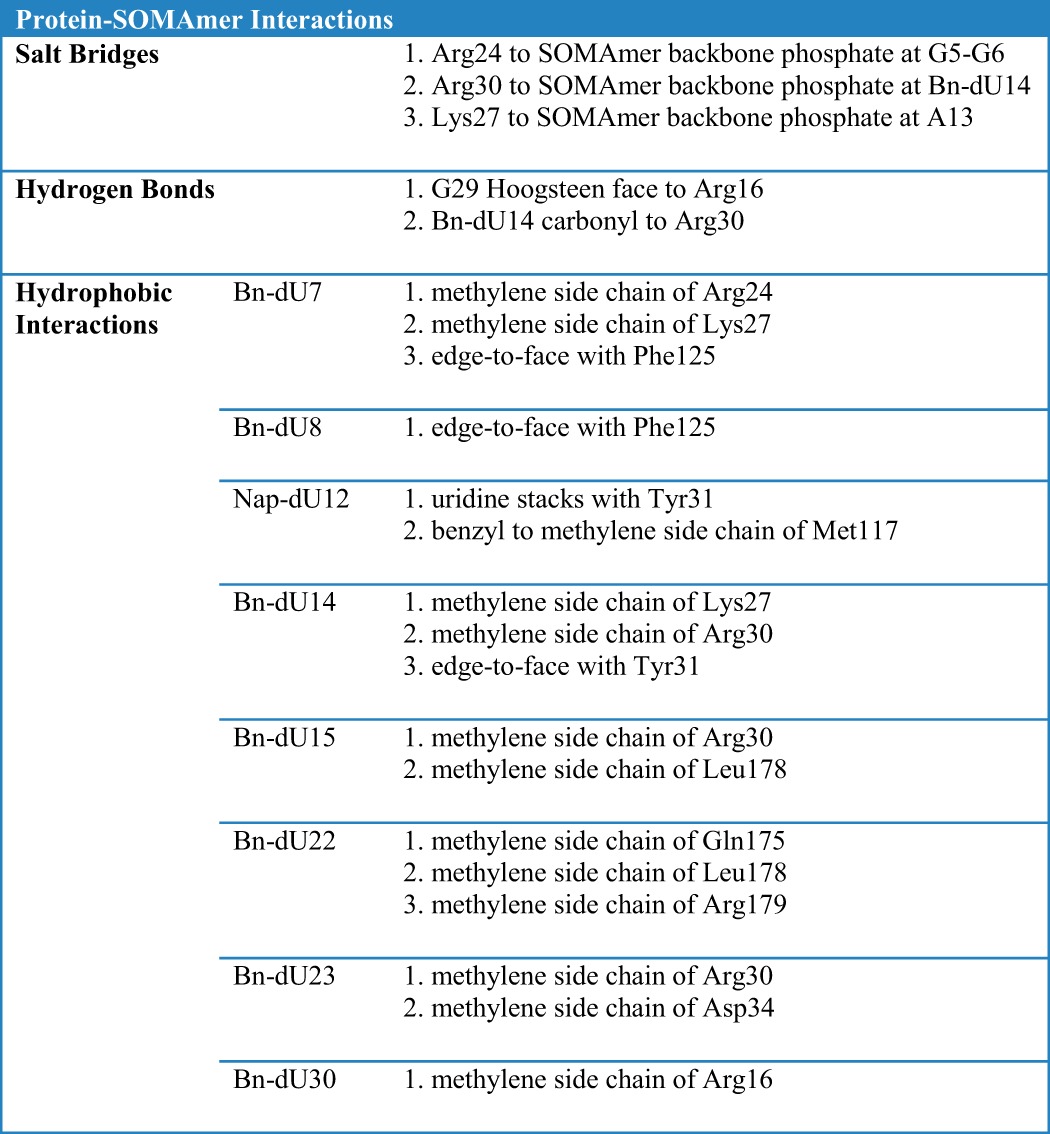
**Complete list of IL-6/SOMAmer interactions**

##### Receptor Mimicry

**FIGURE 7.**
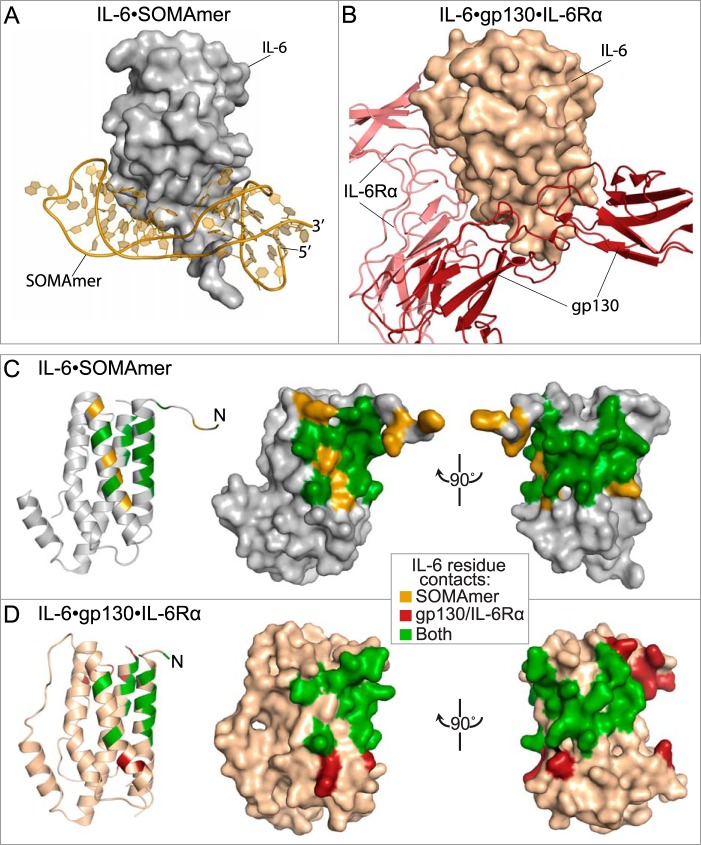
**Overlap of SOMAmer and receptor binding sites on IL-6.** Global views of IL-6 interactions with the SOMAmer (*A*) and the IL-6 receptors IL-6Rα and gp130 (*B*). Comparison of residues on the IL-6 protein within 4 Å of the SOMAmer (*C*) and gp130·IL-6Rα bound (*D*) structures. Residues unique to the SOMAmer structure (*gold*), residues unique to the receptor bound structure (*deep red*), and residues in common to both structures (*green*) are shown in a cartoon and two different orientations of the surface representation.

The binding interface on IL-6 engaged by the SOMAmer overlaps extensively with the regions involved in IL-6 binding to its two cell-surface receptors, IL-6Rα and gp130. Domain 1 of the SOMAmer occupies the binding site exclusively involved in binding to gp130, whereas domain 2 primarily occupies the binding site for IL-6Rα (Fig. 7). The degree to which the SOMAmer engages IL-6 in a manner that resembles the receptors is only partly evident when considering global overlap of the binding surfaces. Consideration of specific interactions illustrates an even greater extent of receptor mimicry.

**FIGURE 8.**
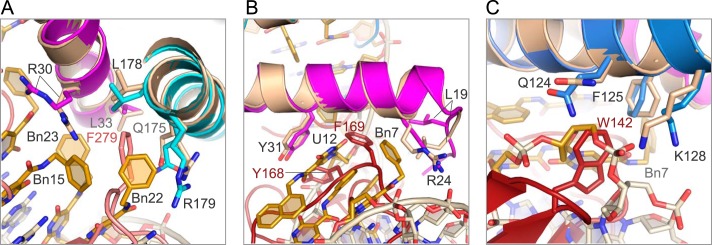
**Detail of SOMAmer and receptor-binding sites.** *A,* residue Phe-279 on IL-6Rα and Bn22 on the SOMAmer (domain 2) recognize the same binding site on IL-6 helices A and D (*pink* denotes IL-6Rα, other colors as in other figures). *B,* SOMAmer (domain 1) and gp130 recognize the same binding site on the IL-6 protein. Phe-169 of gp130 and Bn7, Bn8 and Nap12 of the SOMAmer interact with Tyr-31 and the methylene side chains of Leu-19 and Arg-24 on helix A of IL-6 (*deep red* denotes gp130). *C,* SOMAmer backbone between G6 and Bn7 in the IL-6·SOMAmer structure occupies the same space as Trp-142 in the IL-6·IL-6Rα·gp130 structure.

The hexameric structure of IL-6 bound to the IL-6Rα receptor and the signaling receptor gp130 identified three surfaces on IL-6 involved in protein/protein interactions (15). Site I consists of helices A and D, which interact with IL-6Rα to bury ∼1200 Å^2^. Key residues on IL-6Rα at this interface are Phe-229 and Phe-279. Phe-229 has edgewise interactions with the methylene side chains of Arg-179 and Gln-183 on helix D of IL-6. Phe-279 also interacts with the methylene side chain of Arg-179 on the opposite face and sits in a hydrophobic hollow created by the nonpolar side chains of Arg-179, Gln-175, and Leu-178 on helix D and Leu-33 and Arg-30 on helix A. This is the same hydrophobic pocket occupied by Bn22 within domain 2 of the SOMAmer structure, where the only difference is the rotation of the benzyl group of Bn22 by ∼70° relative to Phe-279 (Fig. 8*A*). There is not a nucleotide in the SOMAmer that interacts on the same surface of IL-6 as Phe-229 of IL-6Rα.

During IL-6 signal transduction, the IL-6·IL-6Rα heterodimer is the first complex to form, followed by gp130 binding to sites IIa on IL-6 and site IIb on IL-6Rα (27, 28). Binding to site IIa also buries ∼1200 Å^2^ and includes helices A and C of IL-6. Residue Phe-169 on gp130 interacts with a surface on helix A containing Leu-19, Arg-24, Lys-27, and Tyr-31, primarily through hydrophobic interactions with nonpolar side chains (15). Many of these residues (Arg-24, Lys-27, and Tyr-31) are also involved in SOMAmer binding to IL-6. Moreover, Phe-169 occupies the same binding pocket in the IL-6·IL-6Rα·gp130 structure as Bn7, Bn8, and Nap12 in the IL-6·SOMAmer structure (Fig. 8*B*). The SOMAmer backbone between G6 and Bn-dU7 occupies the same site as Trp-142 on gp130 (Fig. 8*C*). Trp-142 has an edge-to-face interaction with Phe-125 of IL-6 as well as hydrophobic interactions with the nonpolar side chains of Gln-124 and Lys-128 on helix C (Fig. 8*C*). The second gp130 molecule in the hexameric structure binds to site III on IL-6, which is located at the opposite pole of the four-helix bundle and contains no overlapping binding sites with the SOMAmer (29). The extensive overlap of surfaces on IL-6 engaged by the SOMAmer and the receptors is consistent with the observed ability of the SOMAmer to inhibit IL-6-mediated effects (36).

##### Activity of the G-quartet Domain Fragment and Its Post-SELEX Optimization

**FIGURE 9.**
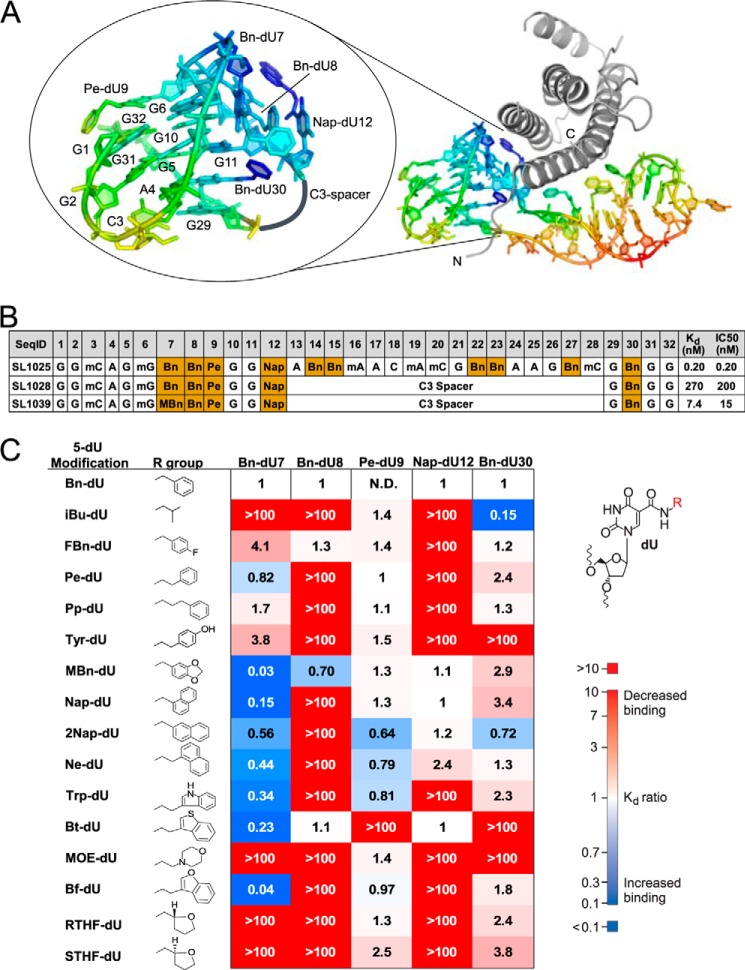
*A,* structure of the G-quartet domain of the IL-6 SOMAmer, depicted with a C3 spacer inserted between Nap-dU12 and G29 to connect the two discontiguous sections of the domain, showing the probable structure of the G-quartet fragment. The SOMAmer is colored by *B*-factors from most ordered (*blue*) to least ordered (*red*) atoms. *B,* sequences and binding affinities of IL-6 SOMAmer (SL1025) and two G-quartet fragments (SL1028 and SL1039) connected by a C3 spacer. *C,* summary of systematic replacement of modified nucleotides in the G-quartet fragment (SL1028). Values shown are the ratio of the *K_d_* values (*K*_*d*_^variant^/*K*_*d*_^parent^). The *K_d_* value for the parent fragment (SL1028) is 2.7 × 10^−7^ m. (*N.D.* = not determined; *Bn-dU*, 5-(*N*-benzylcarboxamide)-2′-deoxyuridine; *iBu-dU,* 5-(*N*-isobutylcarboxamide)-2′-deoxyuridine; *FBn-dU,* 5-[*N*-(4-fluorobenzyl)carboxamide]-2′-deoxyuridine; *Pe-dU,* 5-[*N*-(phenyl-2-ethyl)carboxamide]-2′-deoxyuridine; *Pp-dU,* 5-[*N*-(phenyl-3-propyl)carboxamide]-2′-deoxyuridine; *Tyr-dU,* 5-[*N*-(4-hydroxyphenyl-2-ethyl)carboxamide]-2′-deoxyuridine; *MBn-dU,* 5-[*N*-(3,4-methylenedioxybenzyl)carboxamide]-2′-deoxyuridine; *Nap-dU,* 5-[*N*-(1-naphthylmethyl)carboxamide]-2′-deoxyuridine; *2Nap-dU*, 5-[*N*-(2-naphthylmethyl)carboxamide]-2′-deoxyuridine; *Ne-dU,* 5-[*N*-(1-naphthyl-2-ethyl)carboxamide]-2′-deoxyuridine; *Trp-dU,* 5-[*N*-(3-indole-2-ethyl)carboxamide]-2′-deoxyuridine; *Bt-dU,* 5-[*N*-(3-benzo[*b*]thiophene-2-ethyl)carboxamide]-2′-deoxyuridine; *MOE-dU,* 5-[*N*-(1-morpholino-2-ethyl)carboxamide]-2′-deoxyuridine; *Bf-dU,* 5-[*N*-(3-benzo[*a*]furan-2-ethyl)carboxamide]-2′-deoxyuridine; *RTHF-dU,* 5-[*N*-((*R*)-2-tetrahydrofurylmethyl)carboxamide]-2′-deoxyuridine; *STHF-dU,* 5-[*N*-((*S*)-2-tetrahydrofurylmethyl)carboxamide]-2′-deoxyuridine).

The composite structure of the SOMAmer raises the possibility that the two domains represent separable binding modules, each with sufficient structural integrity to allow it to bind independently to IL-6 with a fraction of the binding affinity of the entire ligand. To test this notion, we synthesized several variants of domains 1 and 2. Fragments representing various forms of the stem-loop domain did not show appreciable binding affinity for IL-6 at protein concentrations up to 5 μm (data not shown). In contrast, a fragment containing the G-quartet domain composed of positions 1–12 and 29–32, with a C3 spacer connecting the two sequence regions (SL1028), exhibited a binding affinity to IL-6 of ∼270 ± 89 nm (Fig. 9, *A* and *B*). This 16-nucleotide fragment corresponds to the entire G-quartet domain, in which the C3 spacer replaces the stem-loop domain that connects the two regions of the G-quartet in the full-length SOMAmer. The binding affinity of this G-quartet fragment is therefore about 1000-fold weaker compared with the full-length SOMAmer. Nevertheless, in terms of free energy of binding, or −Δ*G* values, which is 13.7 kcal/mol for the full-length SOMAmer and 9.3 kcal/mol for the G-quartet fragment, the G-quartet domain appears to make a major contribution to the overall binding affinity of the full-length SOMAmer. This is consistent with the observation that the *B*-factors for domain 1 are generally lower than those for domain 2 (Fig. 9*A*), most likely because the G-quartet provides rigidity to this half of the SOMAmer structure, which helps to maintain its conformational integrity as an isolated domain. The sequence-scrambled analog of the G-quartet fragment (a sequence that maintained the G-quartet but rearranged the positions of the modified nucleotides) showed no binding to IL-6 at protein concentrations up to 1 μm (data not shown).

The 32-mer IL-6 SOMAmer SL1025 demonstrates potent inhibition of IL-6 signaling in cell culture (36). Aside from maintaining a substantial fraction of the binding affinity, the G-quartet fragment SL1028 clearly retains detectable IL-6 inhibitory activity *in vitro* (Fig. 9*B*).

Taking the G-quartet fragment as a new lead, we examined the effect of substituting each of the five modified nucleotides with a collection of alternative 5-position substituents (Fig. 9*C*). Toward this goal, we used a collection of 5-position modifications designed to include privileged fragments of small molecule drugs as well as analogs of amino acid side chains commonly found at protein-protein interfaces. Viewing the G-quartet as a scaffold that orients the side chains of modified nucleotides toward the protein binding interface, we sought to identify functional groups that improve the binding affinity to IL-6 using a strategy typically employed by medicinal chemists. This exercise is also similar to affinity maturation of antibodies, where the initial germ line repertoire within the complementarity determining regions can be refined by hypermutation to further improve binding affinity to an antigen (30, 31). With SOMAmers, the new functional groups are introduced chemically; therefore, as long as a functional group is synthetically accessible, it can be introduced into the SOMAmer in a site-specific manner.

**FIGURE 10.**
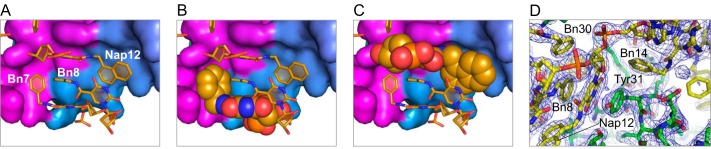
**Bn7 only partly fills the groove on the IL-6 surface, whereas Nap12 completely fills the cavity at the protein·SOMAmer interface.** Packing and shape complementarity at the IL-6·SOMAmer interface are shown in *surface* (IL-6) and *stick* representation (SOMAmer) (*A*) and space-filled renderings of Bn7 (*B*) and Nap12 (*C*). *D,* 2*F_o_* − *F_c_* electron density map contoured at 1.5 σ shows clear density for the aromatic stacking interactions between Tyr-31 on IL-6 and the modified nucleotides of Nap-dU12 and Bn-dU8 on the SOMAmer.

The effect of 15 alternative 5-position substituents introduced at each of the five modified dU residues is summarized in Fig. 9*C*, where the change in affinity from the reference (parent) sequence is expressed as the ratio of dissociation constants (*K_d_* value of variants divided by the *K_d_* value of a reference ligand SL1028). This is a much larger and more diverse set of alternative modifications compared with the four we employed with the full-length ligand (36), reflecting our motivation to achieve a larger affinity improvement. Within the set of 15 alternative moieties, the five modified nucleotide positions of the G-quartet fragment vary considerably with regard to their sensitivity to substitutions. Position 9 was the most tolerant to substitution, with 13 out of 15 replacements being essentially neutral and showing less than a 2-fold effect on binding affinity. This was not unexpected in view of the fact that the modified nucleotide side chain at position 9 is not in contact with the protein and instead is exposed to the solvent. At positions 8 and 12, however, most substitutions were distinctly unfavorable, and none led to an improvement in affinity. Position 30 tolerated a wider variety of substitutions, but only the smaller isobutyl substitution showed an improvement in affinity of about 6-fold. In contrast, position 7 was highly sensitive to modification, with notable affinity changes observed in both favorable and unfavorable directions. Replacement of the aromatic benzyl group with nonaromatic side chains was uniformly unfavorable and led to a reduction in affinity of >100-fold. Replacement with larger aromatic functional groups, however, consistently resulted in affinity improvement. With one such substitution (MBn-dU), affinity improvement of 37-fold was observed. In terms of absolute affinity, this translates to a *K_d_* value of 7.4 ± 6.3 nm (Fig. 9*B*). This represents the largest affinity improvement we have observed to date with a single functional group replacement in a post-SELEX optimization. The improvement in binding affinity is also reflected in the 13-fold enhanced inhibitory activity *in vitro* (Fig. 9*B*). Bn7 partially fills a deep cleft on the surface of IL-6 (Fig. 10). Thus, the advantageous effects of double-ring aromatic substituents can be rationalized by their ability to occupy this pocket more fully. The further improvement wrought by the MBn-dU moiety may reflect its ability to act as a hydrogen bond acceptor for polar groups deep within the pocket, such as the backbone amide NH group of Gln-28. Similarly, the well tolerated double-ring aromatic substituents at position 12 fit snugly into a pocket on the IL-6 surface, providing efficient packing of the interface (Fig. 10). Single-ring aromatics would only partially fill this cavity, accounting for the observed detrimental effects on binding affinity.

## DISCUSSION

The development of new classes of inhibitors aimed at validated targets for pharmacologic intervention is a central component of drug discovery. SOMAmers represent a novel class of binding reagents that combine the advantages of protein-based and nucleic acid-based ligands. We have described a structure of a complex between one such target, IL-6, and a high affinity nucleic acid-based ligand that binds to IL-6 and potently inhibits its biologic activity. The IL-6·SOMAmer co-crystal structure has provided valuable insights into the results from the SELEX experiment with Bn-dU library, including consensus sequence, minimal sequence required for binding, and tolerance to substitutions (36).

The SOMAmer engages IL-6 in a tight fit characterized with extensive shape complementarity. The binding interface of 1248 Å^2^ falls within the range observed for conventional aptamers and is very similar to the two other SOMAmers for which target-bound co-crystal structures have been solved (12). Compared with traditional aptamers, SOMAmers exhibit markedly fewer polar contacts relative to the binding interface area. There are only four H-bonds and five charge/charge interactions for a total of nine polar contacts in the IL-6·SOMAmer structure. In contrast, of the six co-crystal structures with aptamers that bind to their targets with *K_d_* values less than 100 nm, von Willebrand factor, and NF-κB complexes have the most similarly sized interface areas of 1011 and 870 Å^2^. However, the von Willebrand factor complex has 27 polar contacts (13 H-bonds and 14 charge/charge interactions) and NF-κB has 17 polar contacts (five H-bonds and 12 charge/charge interactions) (32, 33). As another comparison, the IgG·aptamer complex has the same number of polar contacts (nine, six H-bonds and three charge/charge interactions) as the IL-6·SOMAmer complex; however, these contacts are packed in an interface area of only 477 Å^2^, which is 38% smaller than that of the IL-6·SOMAmer complex (34). The reduced number of polar contacts in SOMAmers comes without any compromise in binding affinity. In fact, for this admittedly limited set of interactions for which crystal structures are available, SOMAmers show a trend toward higher binding affinities with average free energy of binding, or −Δ*G* value, of 11.4 ± 1.3 kcal/mol for the six aptamers compared with 14.3 ± 0.8 kcal/mol for the three SOMAmers (12).

Taken together, these observations are consistent with the larger contribution to binding from hydrophobic interactions in SOMAmers compared with conventional aptamers. In the IL-6 SOMAmer, of the 10 modified side chains, eight are involved in making contacts with the protein, with the remaining two being solvent-exposed. Therefore, and as we observed previously (12), a high percentage of modified nucleotides are utilized for direct binding interactions with their protein target, illustrating their importance in creating the contact surface. Aside from interacting with the protein, some of the hydrophobic side chains in the G-quartet domain clearly also assist in stabilizing the internal structure of the nucleic acid ligand. We have observed this dual role of modified side chains previously, at the junction between two domains in the PDGF-BB SOMAmer, so this appears to be a recurring phenomenon (12), which is also typical in protein-based ligands. Whether and to what degree these internal hydrophobic cores are maintained as nucleic acid structural elements in the absence of protein ligand remain to be determined.

The IL-6 SOMAmer is comprised of two distinct domains, a G-quartet domain and a stem-loop domain. The existence of two distinct domains was also observed with the PDGF-BB SOMAmer, which has a small pseudoknot (miniknot) domain and a stem-loop domain. Based on truncation studies in which most or all of the stem-loop domain was deleted (leaving only the miniknot domain), binding to PDGF-BB was more than 3 orders of magnitude weaker (*K_d_* = 74 nm) compared with the binding affinity of the complete SOMAmer (*K_d_* = 0.02 nm). The sequence containing the stem-loop domain but lacking an intact miniknot domain had no detectable binding activity. It therefore appears that in the PDGF-BB SOMAmer, most of the binding energy is confined to the miniknot domain (12). In the IL-6 SOMAmer, we have observed a similar phenomenon; the G-quartet domain could be completely disconnected from the full-length ligand and still maintain appreciable, albeit 1000-fold lower, binding affinity to IL-6. Based on free energies of binding (−Δ*G* values of 9.3 kcal/mol for the G-quartet and 13.7 kcal/mol for the full-length ligand), the G-quartet represents about 70% of the binding energy of the full-length ligand. In contrast, the binding affinity of the stem-loop domain appears to be considerably weaker because no binding was detected up to 5 μm. Without a value for the binding affinity of the stem-loop domain alone, we cannot estimate its contribution to the binding affinity of the full-length ligand or any connectivity effects between the two domains (35). Nevertheless, it is clear that both the IL-6 and the PDGF-BB SOMAmers are comprised of two distinct domains, connected through hinge regions, with one primary domain that contributes most of the binding energy and that can be studied independently of the full-length molecule. One major difference between the two structures is in the hinge region, which in the PDGF SOMAmer is highly structured and reinforced by π-stacking interactions among the modified nucleotides, whereas in the IL-6 SOMAmer it appears to be virtually unstructured.

The G-quartet domain fragment can be thought of as a new lead that can be improved independently with post-SELEX optimization. This domain has the lowest *B*-factors in the entire full-length SOMAmer, so it is likely that point mutations we have introduced are made within a fairly rigid scaffold. Of the five modified nucleotides in the G-quartet, position 7 is by far the most sensitive to substitutions. Four out of 15 substitutions result in affinity improvement of at least 5-fold. The 37-fold improvement in binding with a single substitution is the largest improvement we have seen to date. This is accomplished with a relatively small change in the composition of the modified nucleotide (Bn-dU to MBn-dU substitution). The observation that every double-ring modified nucleotide tested at position 7 leads to an improvement in binding affinity can likely be attributed to an increase in buried surface area, more extensive packing at the SOMAmer·IL-6 interface, fortification of the hydrophobic cluster, or the combination of these effects. Conversely, smaller, nonaromatic modifications at this same position that cannot participate in energetically favorable π-stacking and hydrophobic interactions abate binding to IL-6. A similar effect was observed in the PDGF-BB SOMAmer when a nonaromatic isobutyl modification was substituted for a benzyl modification in the middle of a hydrophobic cluster, creating a distinct hole in the middle of the SOMAmer and resulting in reduced binding affinity (12). Further iterative optimization with additional structural variants of the best side chains at this position could result in additional improvement in binding affinity of this diminutive fragment. Improvement in binding affinity by post-SELEX optimization correlates with an improvement in inhibitory activity. This is not surprising, because the G-quartet domain binding site overlaps with that of the signaling gp130 receptor.

The co-crystal structure of the IL-6·SOMAmer complex described in this paper illustrates the profound influence of modified nucleotides on SOMAmer conformation and on composition of the interaction surface in contact with the protein. The two-domain structure of the SOMAmer resembles a clamp that embraces IL-6 with precise shape complementarity encompassing both gp130- and IL-6Rα-binding sites, including extensive receptor mimicry extending all the way to the level of side-chain interactions. Viewing the SOMAmer as an assembly of modular structural elements, we have shown that one of the domains can be separated from its full-length parent and optimized independently, creating the means for fine-tuning the ligand binding surface and therefore specificity. Potent, specific, and chemically versatile synthetic binding reagents of this type have broad potential utility in many areas of pharmaceutical research, including the possibility for development as therapeutics.

## Footnotes

3 The abbreviations used are:

SELEX: systematic evolution of ligands by exponential enrichment
Bn-dU: 5-(*N*-benzylcarboxamide)-2′-deoxyuridine
Nap-dU: 5-(*N*-(1-naphthylmethyl)carboxamide)-2′-deoxyuridine
PDB: Protein Data Bank
SAD: single wavelength anomalous dispersion
Pe-dU: 5-[*N*-(phenyl-2-ethyl)carboxamide]-2′-deoxyuridine
r.m.s.d.: root-mean-square deviation
SAS: solvent-accessible surface area
MBn-dU: 5-[*N*-(3,4-methylenedioxybenzyl)carboxamide]-2′-deoxyuridine
BisTris: 2-[bis(2-hydroxyethyl)amino]-2-(hydroxymethyl)propane-1,3-diol.
